# 

**Published:** 1890-04-26

**Authors:** 


SATURDAY, APRIL 26th, 1890.

**A Hospital Inquisition** - 43

**Words of Consolation.**—The Power of Pain - 44

**The Doctor's Pulpit.**—"For the Complexion": A Rival to Pears - 45

**Doctoring John Chinaman** - 46

**Home and Pay Hospitals.**—Mr. Burdett on the Pay System 47

**Turning Over New Leaves.**—Dr. H. Kuhne's Guide to the Demonstration of Bacteria in the Tissues—Egypt as a Winter Resort—A Manchester Shirt Maker—Influenza and Colds - 48

**Everybody's Page** - 49

**Notes and News** - 50

**Glances at Foreign Asylums** - 57

**Annotations.**—The College of Physicians—The late Bishop Callaway—The Indoor Staff at Stockton - 52

**The Practitioner's Mirror and Retrospect:**

Medicine—Sclerema and Œdema Neonatorum—Constipation 53

Surgery—Examination of the Liver—Gangrene - 53

Hygiene—Scaff's Leather and Rubber Boots - 54

The Practitioner's Bookshelf—The Pulse - 55

**Asylum Construction.**—Description of the New Hospital Wings of the Royal Asylum, Perth - 55

**Working Women in Large Towns.**—I. In England and in America - 56

**Passing Topics.**—Retiring Allowances to Charity Officials - 58

**Scraps and Gleanings** - 58

### EXTRA SUPPLEMENT—THE NURSING MIRROR.

**En Passant.**—The Bicester Home—The Queen's Nurses in Wales—Short Items—News from Kalihi—The Rural Nursing Association—St. John's House—Beef Tea—British Nurses - xiii
**Nursing Lectures.** By Percy Lewis, M.D. IX.—The Kidneys - xiv
**Appointments** - xiv
**The Passing of the Shadow** (*continued*) - xv
**Everybody's Opinion.**—The Junius S. Morgan Memorial—Two Suggestions—Asylum Nurses—Male Nurses - xv
**Notes and Queries. Vacancies** - xvi
**Amusements and Relaxation** - xvi

